# An antagonistic interaction between PlexinB2 and Rnd3 controls RhoA activity and cortical neuron migration

**DOI:** 10.1038/ncomms4405

**Published:** 2014-02-27

**Authors:** Roberta Azzarelli, Emilie Pacary, Ritu Garg, Patricia Garcez, Debbie van den Berg, Philippe Riou, Anne J. Ridley, Roland H. Friedel, Maddy Parsons, François Guillemot

**Affiliations:** 1 Division of Molecular Neurobiology, MRC National Institute for Medical Research, Mill Hill, London NW7 1AA, UK; 2 Randall Division of Cell and Molecular Biophysics, King's College London, London SE1 1UL, UK; 3 Department of Neuroscience, Icahn School of Medicine at Mount Sinai, 1425 Madison Avenue, New York, New York 10029, USA; 4 Present address: Hutchison/MRC Research Centre, University of Cambridge, Box 197, Biomedical Campus, Cambridge CB2 0XZ, UK; 5 Present address: INSERM, Neurocentre Magendie, Physiopathologie de la Plasticité Neuronale, U862, Bordeaux F-33000, France or University Bordeaux, Neurocentre Magendie, Physiopathologie de la plasticité neuronale, U862, Bordeaux F-33000, France; 6 Present address: Protein Phosphorylation Laboratory, Cancer Research UK, London Research Institute, Lincoln's Inn Fields Laboratories, London WC2A 3LY, UK

## Abstract

A transcriptional programme initiated by the proneural factors Neurog2 and Ascl1 controls successive steps of neurogenesis in the embryonic cerebral cortex. Previous work has shown that proneural factors also confer a migratory behaviour to cortical neurons by inducing the expression of the small GTP-binding proteins such as Rnd2 and Rnd3. However, the directionality of radial migration suggests that migrating neurons also respond to extracellular signal-regulated pathways. Here we show that the Plexin B2 receptor interacts physically and functionally with Rnd3 and stimulates RhoA activity in migrating cortical neurons. Plexin B2 competes with p190RhoGAP for binding to Rnd3, thus blocking the Rnd3-mediated inhibition of RhoA and also recruits RhoGEFs to directly stimulate RhoA activity. Thus, an interaction between the cell-extrinsic Plexin signalling pathway and the cell-intrinsic Ascl1-Rnd3 pathway determines the level of RhoA activity appropriate for cortical neuron migration.

## Abstract (web summary)

The small GTPases Rnd2 and Rnd3 act downstream of proneural factors to control the migrating behaviour of neurons in the mouse embryonic cerebral cortex. Here, Azzarelli *et al*. show that Rnd3 binding to the semaphorin receptor PlexinB2 fine-tunes the levels of active RhoA required for cortical neuron migration.

## Graphical abstract

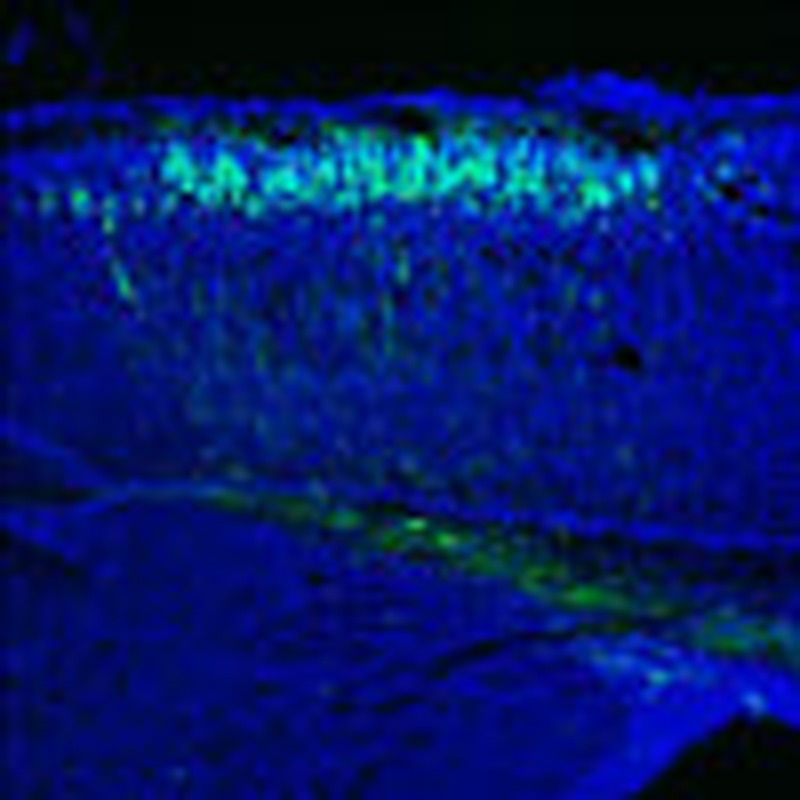

In humans, the cerebral cortex constitutes the site of higher cognitive functions, including memory, attention, perceptual awareness, thought and language. The elaboration of these sophisticated functions relies on appropriate neuronal positioning and connectivity that are predominantly established during embryonic development. In particular, the formation of the mammalian cerebral cortex requires the sequential generation of different neuronal populations and extensive migration of neurons from their germinal zones to their final destination. The importance of neuronal migration during fetal stages for normal functioning of the mature brain is underscored by the fact that multiple neurological disorders, including mental retardation and epilepsy are caused by defects in this specific developmental step^1^,^2^,^3^. Understanding how neuronal migration is controlled during cerebral cortex development is therefore crucial to provide insights into the pathogenesis of many neurodevelopmental cognitive disorders.

During the development of the cerebral cortex, newborn pyramidal neurons undergo radial migration to reach their final position within the cortical plate (CP). Radial migration is a multi-phasic process starting with the detachment of the new neurons from the apical surface of the ventricular zone (VZ)^4^. Neurons then move to the intermediate zone (IZ) where they acquire a multipolar shape and actively extend and retract dynamic processes. After sojourning in the IZ, neurons enter the CP where they become bipolar, extending a leading process towards the pial surface and a trailing process in the opposite direction. During this phase, cells migrate towards the upper part of the CP using glia-guided locomotion, a mode of migration characterized by repetitive migratory cycles of extension of the leading process, translocation of the nucleus and retraction of the trailing process^5^. As neurons approach their destination, they change again their mode of migration from locomotion to terminal translocation^6^ and finally settle in a specific cortical layer.

The precise orchestration of radial migration of cortical neurons depends on both intracellular and extracellular cues^7^,^8^. We have shown previously that two small GTP-binding proteins, Rnd2 and Rnd3, have crucial roles in different phases of cortical neuron migration through inhibition of RhoA signalling in different subcellular locations. Rnd2 controls the transition from the multipolar to the bipolar stage and the extension of the leading process while Rnd3 regulates locomotion^9^,^10^. Rnd2 and Rnd3 proteins are regulated at the transcriptional level by the proneural factors Neurog2 and Ascl1, respectively. In addition to such intrinsic regulators of neuronal motility, a huge variety of secreted and membrane-bound extracellular molecules have been shown to orchestrate cortical neuron migration, by regulating the motility or the guidance of neurons^11^. Although the function of guidance molecules has been mostly characterized in tangentially migrating cortical interneurons, recently the Semaphorin family member Sema3A and the Netrin receptor Unc5D have been reported to direct radial migration of pyramidal neurons^12^,^13^. Moreover, the Semaphorin receptor, Plexin B2, has been implicated in various aspects of cortical development, although its specific contribution to cortical neuron migration has been difficult to address because of earlier defects in *Plexin B2* mutant cortices^14^.

In spite of these advances, little is known about the signalling machinery that mediates the response of radially migrating neurons to extrinsic cues. Although intrinsic and extrinsic mechanisms regulating neuronal migration have been extensively studied separately, it remains unknown how these two types of pathways are integrated to precisely control the migratory behaviour of cortical neurons. To address this issue, we have examined whether Rnd proteins interact with pathways initiated by extracellular signals. We chose to focus on the interaction between Rnds and Plexin B receptors because of the previous description of Rnd1 involvement in Plexin B1 signalling in cultured cells^15^,^16^. However, functional interaction between Plexin B molecules and Rnd members other than Rnd1 has not been addressed yet.

In the present study, we combine genetic, biochemical and molecular approaches to investigate whether the Ascl1-Rnd3 axis promotes cortical neuron migration via regulation of Plexin B signalling. We provide evidence for a Plexin B2-Rnd3 antagonistic interaction that promotes radial migration of cortical neurons through fine regulation of RhoA activity. We propose therefore that Rnd3 may constitute a hub where an intrinsic programme, driven by the proneural factor Ascl1, and an extrinsic signal, represented by the Plexin-Semaphorin pathway, meet to coordinate neuronal migration.

## Results

### Plexin B2 is required for cortical neuron migration

**Figure 1:**
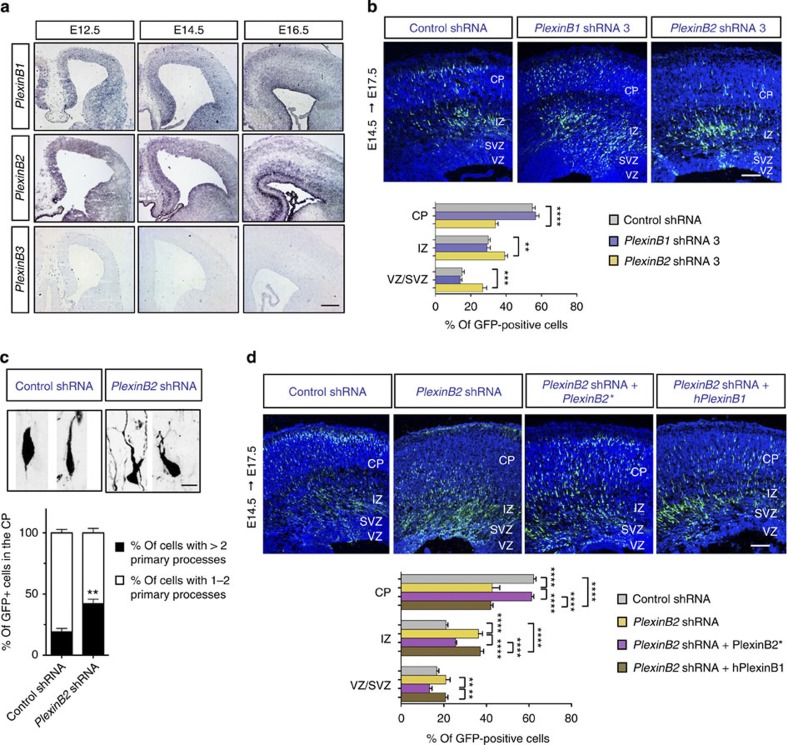
Plexin B2 regulates the migration and the morphology of cortical neurons. (**a**) Distribution of *Plexin B1*, *Plexin B2* and *Plexin B3* transcripts in coronal sections from E12.5, E14.5 and E16.5 mouse brains. Scale bar, 250 μm. (**b**) Migration analysis of cortical neurons electroporated *in utero* with *Plexin B1* or *Plexin B2* shRNAs at E14.5 and analysed 3 days later. Most *Plexin B2*-silenced neurons failed to reach the upper layers of the cortex in comparison with *Plexin B1* shRNA or control-treated neurons. TOTO-3 was used to label nuclei and subdivide the cortical wall into cortical plate (CP), IZ and subventricular zone/ventricular zone (SVZ/VZ). The quantification graph shows the distribution of GFP-positive cells in different zones of the cortex (VZ/SVZ, IZ and CP) in the different electroporation experiments. Data are presented as the mean±s.e.m. from six sections prepared from three embryos obtained from two or three litters. One-way ANOVA followed by the Bonferroni *post-hoc* test; **P<0.01, ***P<0.001, ****P<0.0001. Scale bar, 200 μm. (**c**) Morphology of electroporated cells in the CP. Many *Plexin B2*-silenced neurons displayed abnormal morphologies, characterized by a branch-leading process or supernumerary primary processes. Scale bar, 10 μm. Quantification graph shows the proportion of cells exhibiting more than two primary processes (in black); *n*>150 cells from three different brains. Student’s *t*-test; **P<0.01 compared with control. (**d**) The radial migration defect of *PlexinB2*-deficient neurons was rescued by overexpression of a shRNA-resistant version of *Plexin B2* (Plexin B2*), but not by overexpression of human *Plexin B1* (*hPlexinB1*). Mean±s.e.m. from six sections prepared from three different experiments; one-way ANOVA followed by the Bonferroni *post hoc* test; **P<0.01, ****P<0.0001. Scale bar: 200 μm.

To determine whether Rnd proteins functionally interact with Plexin B receptors in migrating cortical neurons, we begun by examining the expression and function of members of the Plexin B family in the murine embryonic cortex (Fig. 1a). RNA *in situ* hybridization of sections of cerebral cortex at different developmental stages with probes for members of the *Plexin B* family revealed that *Plexin B1* and *Plexin B2* had very similar expression patterns in the VZ, subventricular zone (SVZ) and CP, whereas *Plexin B3* was not expressed in this tissue. *Plexin B1* and *B2* expression also resembled that of *Rnd3* (but not that of *Rnd2*, Supplementary Fig. 1a), suggesting a possible functional relationship between these genes.

To determine whether *Plexin B1* and *Plexin B2* regulate neuronal migration, such as *Rnd3,* we silenced each gene by *in utero* electroporation of short hairpin RNA (shRNA) vectors^10^,^17^. Constructs that efficiently reduced the expression of endogenous *Plexin B1* and *Plexin B2* in P19 cells (Supplementary Fig. 1b,c) were electroporated in the cortex of E14.5 mouse embryos. Analysis of electroporated cortices 3 days later (E17.5) revealed that silencing *Plexin B2* profoundly affected the migration of electroporated neurons, whereas silencing *Plexin B1* had no detectable effect (Fig. 1b). Quantification of the distribution of electroporated cells in the different zones of the cortex showed that more neurons remained in the VZ/SVZ and the IZ, and fewer reached the CP when *Plexin B2* was silenced than in control experiments (Fig. 1b). Moreover, many *Plexin B2*-silenced neurons that had reached the CP had abnormal morphologies, including a forked leading process and/or multiple processes sprouting from the cell body (42.1±3.7%; *n*>150 cells from three different experiments; Fig. 1c), whereas the vast majority of CP neurons in control experiments were bipolar with a characteristic thick leading process and thin trailing process (81.0±3.0%; *n*>150 cells from three different experiments; Fig. 1c). Interestingly, similar morphological anomalies were seen in *Rnd3*-silenced neurons that had reached the CP^10^. Co-electroporation of a knockdown-resistant version of *Plexin B2* (marked Plexin B2*; Supplementary Figs 1d and 6) together with the *Plexin B2* shRNA fully rescued the migratory deficit of *Plexin B2*-silenced cells, thus demonstrating the specificity of this phenotype (Fig. 1d). Co-electroporation of the related receptor *Plexin B1* with the *Plexin B2* shRNA, resulted in a cell migration phenotype indistinguishable from that of *Plexin B2* shRNA alone (Fig. 1d and Supplementary Fig. 1e shows that the *Plexin B2* shRNA does not silence *Plexin B1*), demonstrating that *Plexin B1* cannot compensate for the reduction of *Plexin B2* function, and therefore the two molecules have divergent activities in migrating neurons.

### Plexin B2 and Rnd3 functionally interact in migrating neurons

**Figure 2:**
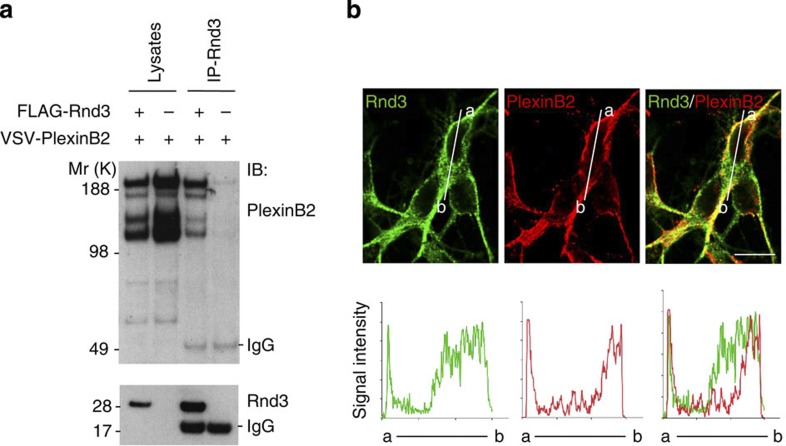
Plexin B2 and Rnd3 interact biochemically. (**a**) Rnd3 co-immunoprecipitates with Plexin B2. P19 cells were transfected with *VSV-Plexin B2* alone or in combination with *FLAG-Rnd3* as indicated. The lysates were immunoprecipitated with anti-FLAG antibody and immunoblotted with anti-VSV or anti-FLAG antibodies. For full blots see Supplementary Fig. 6. (**b**) Distribution of Plexin B2 and Rnd3 proteins in cortical neurons dissociated at E14.5 and cultured for 4 days. Plexin B2 (in red) and Rnd3 (in green) co-localize at the plasma membrane, as shown by the intensity profile along the a–b line. Scale bar, 10 μm.

The involvement of both *Rnd3* (ref. 10) and *Plexin B2* (Fig. 1) in the migration of cortical neurons raised the possibility that the two molecules act together to promote neuronal migration. To examine this idea, we first asked whether the two proteins interact physically by performing co-immunoprecipitation experiments. By immunoprecipitating FLAG-tagged Rnd3 with a FLAG antibody, we were able to detect VSV-tagged Plexin B2 in the co-immunoprecipitated material, demonstrating that the two proteins are able to form a complex (Fig. 2a). The specificity of this interaction is demonstrated by the fact that the related receptor Plexin B1 does not interact with Rnd3 (Supplementary Fig. 1f). Moreover, endogenous Rnd3 and Plexin B2 co-localize at the plasma membrane in acutely dissociated embryonic cortical neurons, suggesting that the two molecules might also interact in cortical cells *in vivo* (Fig. 2b).

**Figure 3:**
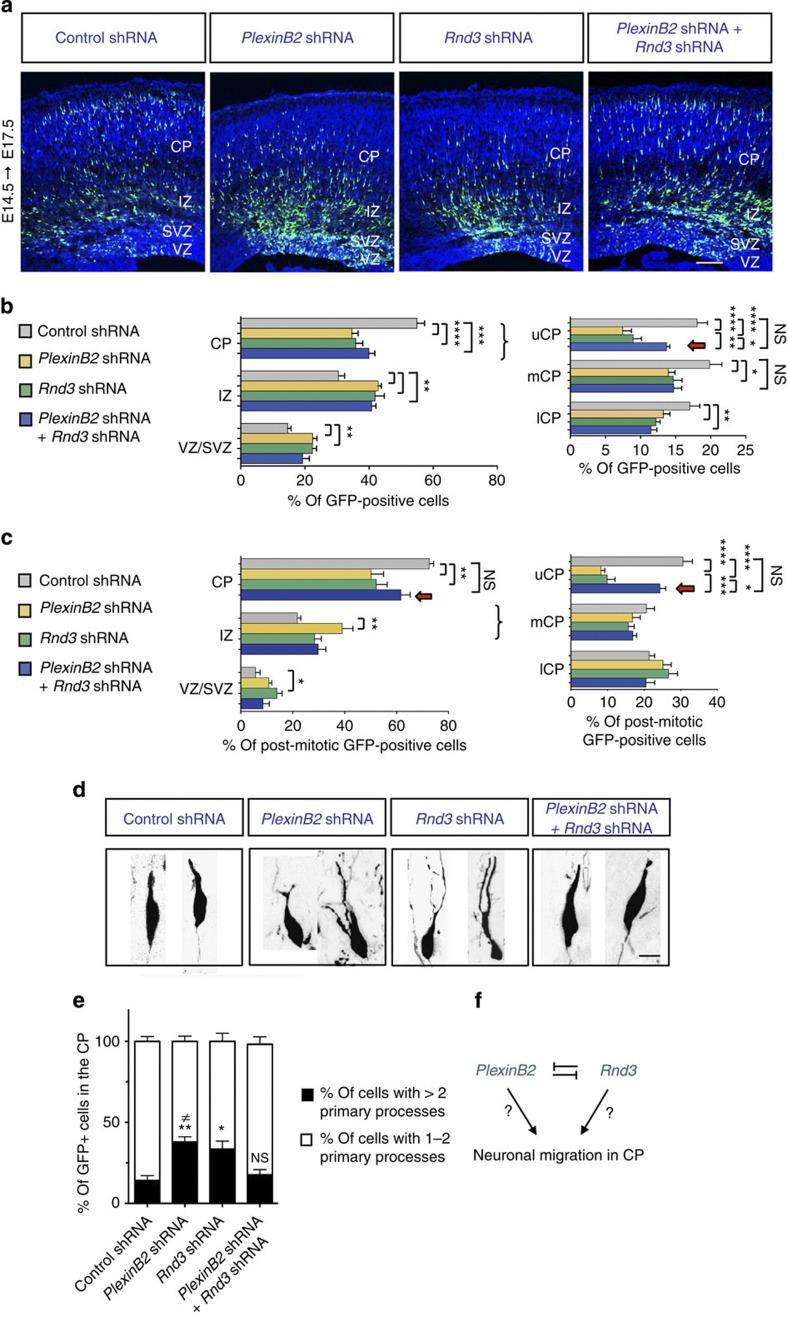
Plexin B2 and Rnd3 antagonize each other’s activities in radially migrating neurons. (**a**) Mouse embryonic cortices electroporated with control shRNA, *Plexin B2* shRNA, *Rnd3* shRNA or *Plexin B2* and *Rnd3* shRNAs together at E14.5 and analysed 3 days later. The migration defect produced by *Rnd3* or *Plexin B2* silencing was partially rescued by the concomitant knockdown of the two genes. Scale bar, 200 μm. (**b**) The quantification graph compares the distribution of GFP+ cells in the different electroporation experiments. The CP is further subdivided into lower CP (lCP), median CP (mCP) and upper CP (uCP). The red arrow in the quantification graph highlights the migratory rescue in the uCP. Mean±s.e.m. from six sections prepared from three different experiments; one-way ANOVA followed by the Bonferroni *post hoc* test; *P<0.05, **P<0.01, ***P<0.001 and ****P<0.0001; NS, not significant. (**c**) The graph reports the distribution of post-mitotic (GFP+/ki67−) cells in the different experiments. The red arrows indicate the migratory rescue when both *Plexin B2* and *Rnd3* are silenced. Mean±s.e.m. from six sections prepared from three different experiments; one-way ANOVA followed by the Bonferroni *post hoc* test; *P<0.05, **P<0.01, ***P<0.001 and ****P<0.0001; NS, not significant. (**d**) Morphology of radially migrating neurons electroporated with the different constructs as indicated. Aberrant shapes were observed in both *Plexin B2*-silenced neurons and *Rnd3*-silenced down cells. Scale bar, 10 μm. (**e**) Quantification of the morphologies of electroporated cells in the CP. The percentages represent the proportion of cells exhibiting more than two primary processes (that is, attached to the cell body). *n*>150 cells from three different brains. One-way ANOVA followed by the Bonferroni *post hoc* test; *P<0.05 and **P<0.01 compared with control, ^≠^*P*<0.05 compared with double knockdown. (**f**) Drawing of the genetic interaction between *Plexin B2* and *Rnd3*. The two factors antagonize each other’s activities in the control of neuronal migration and morphology in the CP.

Next we asked whether the two genes cooperate in cortical neurons to promote the acquisition of proper morphology and cell migration. To this end, we silenced both genes simultaneously by co-electroporating the two shRNA constructs in the murine embryonic cortex. If the two genes function synergistically, combining the two shRNAs should exacerbate the migratory phenotypes produced by each shRNA separately. However, the double knockdown resulted instead in a significant improvement of the migration defects of single knockdown cells, with 13.7±0.5% double knockdown cells reaching the upper part of the CP compared with 7.5±1.2% and 9.0±1.2% single *Plexin B2-* and *Rnd3-*silenced cells, respectively, and 18.1±1.5% cells in the control experiment (Fig. 3a–c), indicating that *Rnd3* and *Plexin B2* antagonize each other during cell migration^18^. The antagonistic interaction with *Plexin B2* was specific to *Rnd3*, as co-electroporation of shRNAs for *Plexin B2* and *Rnd2* did not produce any improvement of the individual migration phenotypes (Supplementary Fig. 2), even if Rnd2 is capable of associating with PlexinB2 in a different context (Supplementary Fig. 1f).

Interestingly, simultaneous knockdown of *Plexin B2* and *Rnd3* rescued the migration defects of single knockdown cells only in the CP, as double knockdown cells accumulated in the VZ/SVZ and IZ to the same extent as single knockdown cells (Fig. 3a,b). This suggests that *Plexin B2* and *Rnd3* display antagonistic activities specifically in neurons undergoing locomotion in the CP, while they function independently of one another before the onset of neuronal migration in the VZ/SVZ and/or during early phases of migration in the IZ. In support of the idea that *Plexin B2* and *Rnd3* antagonize each other in the CP, the aberrant morphological features observed in the CP in *Plexin B2*-silenced neurons and in *Rnd3*-silenced neurons were fully corrected by silencing both genes (Fig. 3d,e). Thus, functional antagonism between *Plexin B2* and *Rnd3* has an important role in regulating the activity of the two molecules in neurons that have reached the CP (Fig. 3f).

In contrast, the accumulation of *Rnd3*-silenced cells in the VZ/SVZ and IZ could be attributed mostly to the recently reported role of *Rnd3* of suppressing the proliferation of basal cortical progenitors through inhibition of the translation of the cell cycle regulator Ccnd1 (ref. 19). As already shown, silencing *Rnd3* resulted in excessive proliferation of basal progenitors, leading to an accumulation of proliferating cells in the VZ/SVZ and IZ and a delay in cell cycle exit and subsequent migration of post-mitotic cells to the CP (Supplementary Fig. 3e,g). Silencing *Plexin B2* alone had no significant effect on progenitor proliferation (Supplementary Fig. 3a,b,d,g), thus explaining the lack of rescue of the accumulation of *Rnd3*-silenced cells in the VZ/SVZ and IZ by co-electroporation of *Plexin B2* shRNA (Supplementary Fig. 3).

### Plexin B2 activates RhoA in part by inhibiting Rnd3

**Figure 4:**
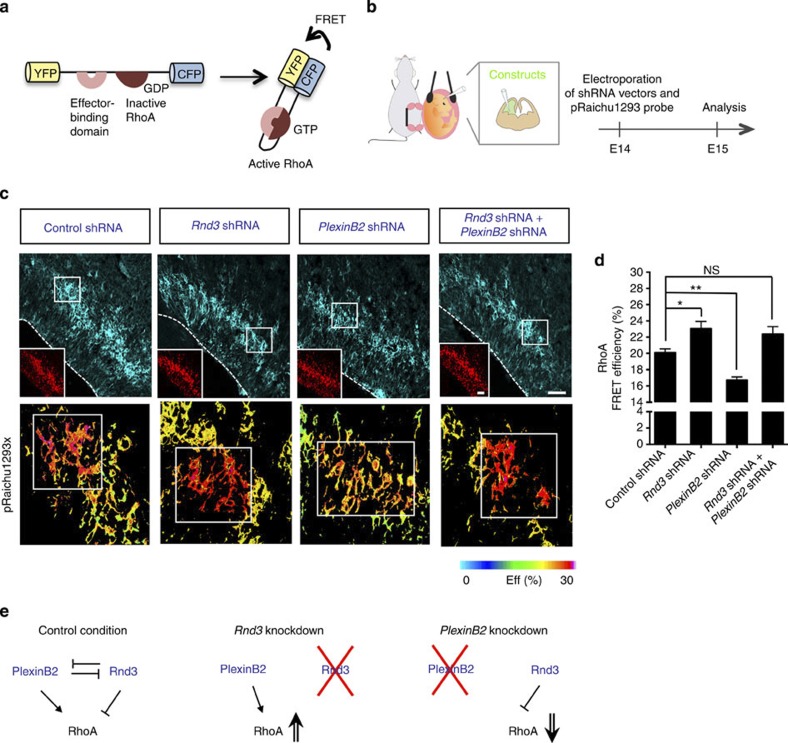
Plexin B2 activates RhoA in migrating neurons in part by inhibiting Rnd3. (**a**,**b**) Schematic representation of the experimental approach. An intra-molecular FRET probe (pRaichu1293) was used to measure the levels of active (GTP-bound) RhoA at the cell membrane (**a**). The probe and shRNA constructs were co-electroporated *in utero* at E14.5 and electroporated cells were analysed 1 day later in brain slices (**b**). (**c**) *In vivo* FRET analysis of RhoA activity performed on cortical slices electroporated with different constructs as indicated. Upper panels show the CFP signal from the FRET probe (scale bar, 50 μm); the RFP signal in insets marks electroporated cells (scale bar in insets, 50 μm). Lower panels show FRET efficiency in the indicated area (white rectangles indicate the bleached area). (**d**) Quantification graph showing the level of FRET signals in electroporated cortical cells in the different conditions. Mean±s.e.m.; one-way ANOVA followed by the Bonferroni *post hoc* test; *P<0.05, **P<0.01; NS, not significant. *n*>12 areas for each condition, deriving from at least three different embryos from three different litters. (**e**) Model of RhoA regulation by Plexin B2 and/or Rnd3 in the different experiments.

The foregoing results showed that *Plexin B2* and *Rnd3* are both required for the migration of CP neurons but that their activities are mutually antagonistic. To determine the possible cause of this apparent paradox, we examined the mechanism underlying the interaction between these two molecules. Similar to Rnd3 (ref. 10), Plexin B receptors have been shown to act in cultured cells through regulation of RhoA signalling, with different studies finding that these receptors either increase^16^,^20^,^21^,^22^,^23^ or reduce^24^ RhoA activity. We therefore asked whether Plexin B2 acts in migrating cortical neurons by regulating activation of RhoA, either alone or in concert with Rnd3. Active RhoA levels at the cell membrane were measured in intact cells *in vivo* by co-electroporating shRNA and a RhoA fluorescence resonance energy transfer (FRET) biosensor in the cortex of E14.5 embryos^25^,^26^. FRET analysis was subsequently performed in fixed brain slices 1 day after electroporation (Fig. 4a,b; refs 10, 26). RhoA activity was detected in cells in the upper IZ and lower CP, and this activity was enhanced by *Rnd3* silencing, as previously reported (Fig. 4c,d; ref. 10). In contrast, RhoA activity was significantly decreased when *Plexin B2* was silenced, indicating that *Plexin B2* positively regulates RhoA activity in migrating cortical neurons (Fig. 4c,d).

**Figure 5:**
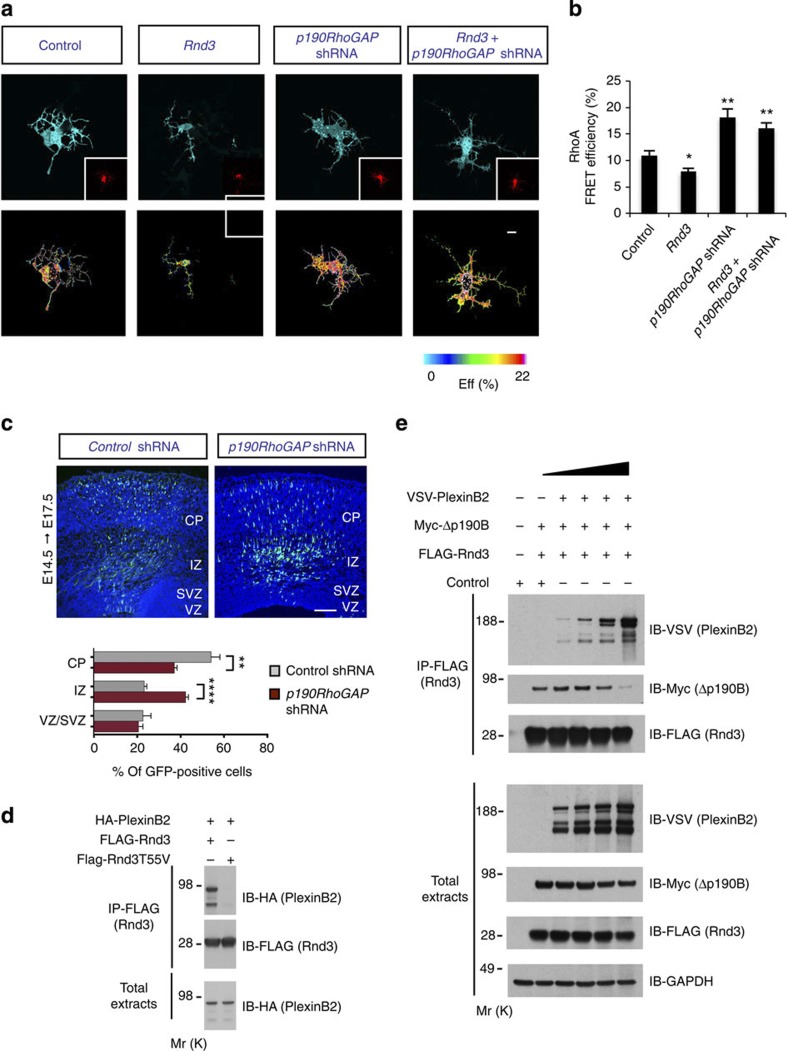
p190RhoGAP mediates Rnd3 inhibitory function towards RhoA and competes with Plexin B2 for Rnd3 binding. (**a**) *In vitro* FRET analysis of RhoA activity in dissociated cortical cells in culture, 2 days after the electroporation of the constructs indicated. Upper panels show the CFP signal from the FRET probe; the RFP signal (in insets) marks electroporated cells (scale bar in insets, 10 μm). Lower panels show FRET efficiency. Scale bar, 10 μm. (**b**) Mean±s.e.m.; (*n*>11 cells for each condition, from three independent experiments; *t*-test: *P<0.05 and **P<0.01 compared with control). (**c**) Mouse embryonic cortices electroporated *in utero* with control shRNA or *p190RhoGAP* shRNA at E14.5 and analysed 3 days later. Scale bar, 200 μm. The distribution of GFP^+^ cells in the different cortical compartments revealed defects in the migration of *p190RhoGAP*-depleted neurons. Mean±s.e.m. from six sections prepared from three different experiments; *t*-test; **P<0.01 and ****P<0.0001. (**d**) Plexin B2 interacts with the same site as p190RhoGAP on Rnd3 protein. COS7 cells were co-transfected with *HA-PlexinB2* (cytoplasmic domain) and wild-type and mutated (T55V) *FLAG*-*Rnd3* constructs. The lysates were immunoprecipitated (IP) with anti-FLAG antibody and immunoblotted with anti-HA or anti-FLAG antibodies. The mutation of a single residue, Threonine 55 in the effector binding domain, of Rnd3 disrupted the interaction with Plexin B2, similar to p190RhoGAP^4^. (**e**) Competition between Plexin B2 and p190RhoGAP for Rnd3 binding. Expression vectors encoding FLAG-Rnd3 and Myc-p190RhoGAP-B (middle domain) were co-transfected with increasing amounts of *VSV-Plexin B2* into COS7 cells. Cell lysates were IP with anti-FLAG antibody, then immunoblotted with the indicated antibodies. For full blots see Supplementary Fig. 6.

As Plexin B2 and Rnd3 antagonize each other’s functions in migrating neurons, we reasoned that these molecules might mutually suppress each other’s activity of regulation of RhoA (Fig. 4e). To test this possibility, we co-electroporated the RhoA FRET probe with both *Plexin B2* and *Rnd3* shRNAs. When both genes were silenced, RhoA activity returned to a level not significantly different from that found in control experiments, suggesting that the improvement in migration of double knockdown neurons can be partially attributed to the correction of RhoA activity (Fig. 4d). Moreover, the restored level of active RhoA seen in *PlexinB2*-silenced neurons when *Rnd3* was also depleted suggests that Plexin B2 activates RhoA by inhibiting Rnd3. The predominant mechanism by which Rnd3 suppresses RhoA activity is by interacting with the RhoA GTPase-activating protein p190RhoGAP^27^. Indeed, the inactivation of RhoA by Rnd3 overexpression is reverted in the absence of p190RhoGAP (Fig. 5a,b and Supplementary Fig. 4a). As expected, p190RhoGAP silencing in cultured neurons results in a strong increase of RhoA activity (Fig. 5a,b), and its silencing *in vivo* induces migration defects that can be attribute to RhoA hyperactivity (Fig. 5c).

We have previously shown that Rnd3 interacts with p190RhoGAP via its Thr55 residue^10^,^27^. Interestingly, mutation of Thr55 to a Val residue disrupts interactions not only with p190RhoGAP but also with Plexin B2 (Fig. 5d), suggesting that Plexin B2 and p190RhoGAP might bind Rnd3 in a mutually exclusive manner. To test this model, we performed competitive co-immunoprecipitation experiments with Plexin B2, p190RhoGAP and Rnd3 co-expressed at different levels. We found that increasing Plexin B2 concentrations resulted in a decrease in p190RhoGAP binding to Rnd3 (Fig. 5e), suggesting that Plexin B2 competes with p190RhoGAP for Rnd3 binding. The disruption of the Rnd3-p190RhoGAP interaction could account at least in part for the activation of RhoA by Plexin B2.

An interaction between PlexinB2 and Rnd3 has been shown to regulate R-Ras signalling during axon guidance^15^. To determine whether the PlexinB2-Rnd3 interaction in cortical neurons also controls R-Ras activity, we performed FRET measurements with an intra-molecular FRET probe for R-Ras (pRaichu 205x) (ref. 28). Silencing *PlexinB2* resulted in an increase in R-Ras activity but no change was observed when *Rnd3* was silenced (Supplementary Fig. 4b). This result indicates that the inhibition of R-Ras activation by the intrinsic R-Ras GAP activity of Plexin B2 is independent of Rnd3, further supporting the conclusion that PlexinB2 and Rnd3 antagonistic functions in migrating cortical neurons predominantly fine-tune the activity level of RhoA.

### Plexin B2 activates RhoA in part by recruiting RhoGEF

**Figure 6:**
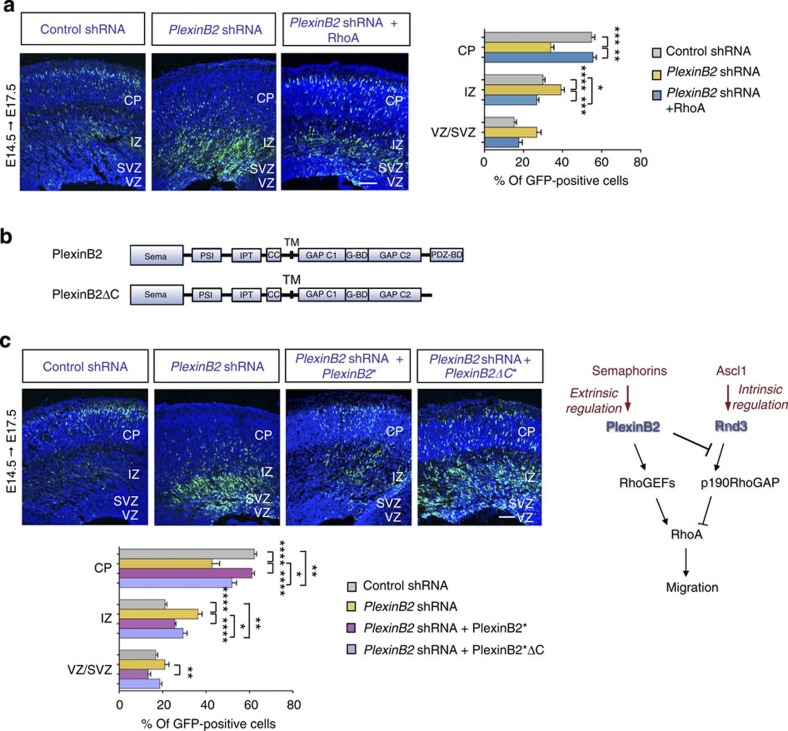
Plexin B2 activates RhoA in the cortex in part by recruiting RhoGEFs. (**a**) The migration defect induced by Plexin B2 shRNA electroporation was rescued by co-electroporation of a RhoA expression vector. The graph shows the distribution of electroporated GFP-positive cells per cortical compartment in the different conditions. Mean±s.e.m. from six sections prepared from three different experiments; one-way ANOVA followed by the Bonferroni *post-hoc* test; *P<0.05, **P<0.01, ***P<0.001 and ****P<0.0001. Scale bar, 200 μm. (**b**) Schematic representation of the different domains of the Plexin B2 protein. The PDZ-binding domain (PDZ-BD) at the C terminus of wild-type Plexin B2 has been removed in the Plexin B2 C-terminal deletion mutant (PlexinB2ΔC). Sema: Sema domain; PSI: plexin, semaphorin and integrin domain; IPT: Ig-like, plexin and transcritpion factor domain; CC: convertase cleavage site; TM: transmembrane domain; GAP C1/C2: segmented GTPase activating protein (GAP) domain; G-BD: GTPase binding domain; PDZ-BD: PDZ-binding domain. (**c**) Images of electroporated cortices and quantification graph show that PlexinB2ΔC* ameliorates the defects induced by Plexin B2 knockdown, although not as efficiently as wild type PlexinB2*. The star (*) indicates that the constructs carry mutations conferring RNAi resistance. Mean±s.e.m. from six sections prepared from three different experiments; one-way ANOVA followed by the Bonferroni *post hoc* test; *P<0.05, **P<0.01 and ****P<0.0001. Scale bar, 200 μm. (**d**) Model of how the Ascl1-Rnd3 and Semaphorin-PlexinB2 interact to regulate RhoA activity in cortical neuronal migration. On the one hand, Ascl1-Rnd3 maintains low background levels of RhoA activity by interacting with p190RhoGAP. On the other hand, upon extracellular activation, Plexin B2 promotes RhoA activation by two mechanisms, blocking Rnd3 interaction with p190RhoGAP and directly recruiting RhoGEFs.

The correction by *Rnd3* knockdown of both the migration defect and the low level of RhoA activity of *Plexin B2*-silenced neurons suggested that this migratory phenotype is due to reduced RhoA activity. However, there is currently little *in vivo* evidence, demonstrating that cortical neurons require a minimum level of RhoA activity to migrate. To determine whether the cell migration defect of *Plexin B2*-silenced neurons could indeed be attributed to lower level of RhoA activity, we examined the effect of increasing RhoA level in these cells. When *Plexin B2* shRNA was co-electroporated with a *RhoA* expression vector, the migration defect of *Plexin B2*-silenced neurons was fully rescued (Fig. 6a). Therefore, RhoA activity is required for the correct migration of cortical neurons, and Plexin B2 promotes cell migration by maintaining an appropriate level of RhoA activity in neurons.

Besides interacting with Rnd3, Plexin B2 has previously been shown to promote axon guidance and growth cone collapse by activating RhoA through direct recruitment of PDZ domain-containing Rho GTP exchange factors (RhoGEFs)^16^,^23^. We thus asked whether the role of Plexin B2 in cortical neuron migration also involves interactions with RhoGEFs. We tested the capacity of a C-terminal deletion mutant of Plexin B2 (PlexinB2 ΔC), which has lost its capacity to interact with RhoGEFs^22^,^23^, to rescue the migratory phenotype of *Plexin B2*-silenced neurons (Fig. 6b,c). Although co-electroporation of the *Plexin B2* shRNA and the knockdown-resistant form of *Plexin B2* fully reverted the silencing phenotype, co-electroporation of a knockdown-resistant form of the C-terminal deletion mutant ameliorated the phenotype only partially (42.7±3.5% neurons reached the CP when Plexin B2 shRNA alone was electroporated, 61.2±1.0% with Plexin B2 shRNA+Plexin B2* and 51.9±2.1% with Plexin B2 shRNA+Plexin B2 ΔC*; *n*=6 sections from three different experiments) (Fig. 6c). When we tested the ability of Plexin B2 ΔC to activate RhoA *in vivo* or in cultured neurons exposed to the Plexin B2 extracellular ligand Semaphorin 4D, we found that Plexin B2 ΔC no longer promoted RhoA activation, whereas full-length Plexin B2 did (Supplementary Fig. 5a–d). Semaphorin-mediated Plexin B2 recruitment of RhoGEFs is therefore essential for local activation of RhoA and correct neuronal migration. Altogether, our data suggest that, as well as suppressing Rnd3 activity, PlexinB2 also stimulates RhoA activation and promotes the migration of cortical neurons by recruiting RhoGEFs (Fig. 6d).

## Discussion

In this study, we have investigated how different pro-migratory signals are integrated at a molecular level to coordinate the multistep process of radial migration of cortical projection neurons. Recent studies have highlighted the importance of transcriptional mechanisms in the regulation of neuronal migration^29^,^30^. In particular, transcriptional control of Rnd proteins is crucial to regulate cytoskeleton dynamics in radially migrating cortical neurons^9^,^10^,^19^,^31^. However, how this cell-intrinsic control of the cytoskeleton interfaces with extracellular signal-regulated pathways that orient the migration of neurons has remained unclear. Here we show that the Ascl1 target Rnd3 and the axon guidance receptor Plexin B2 antagonize each other’s activity to regulate RhoA activity and promote cortical neuron migration.

Rnd1 has previously been implicated in the signal transduction pathway activated downstream of the Plexin B1 receptor during growth cone collapse and inhibition of axon extension^15^. Our results extend this finding by showing that the interaction between Plexin B2 and Rnd3 has a major role in the control of cortical neuron migration *in vivo*. However, we have identified a major difference from previous models. Although the binding of Rnd1 to Plexin B1 intracellular domain has been shown to open the conformation of the receptor and to allow the transmission of the downstream signal in a synergistic manner^32^,^33^, our genetic and molecular data indicate instead that Plexin B2 and Rnd3 antagonize each other’s activity. This is reminiscent of other situations where pairs of genes oppose each other when regulating cell migration and the cytoskeleton. For example, the genes *Dcx* and *Mark2* exhibit antagonistic activities in the control of microtubule stability in migrating neurons, so that cortical neurons fail to migrate properly when either gene is inactivated, whereas simultaneous inactivation of both genes corrects the migration defects^18^. Rnd3 controls actin polymerization in migrating neurons in part via regulation of cofilin^10^. The opposite regulation of RhoA activity by PlexinB2 and Rnd3 is therefore likely to influence the organization of the actin cytoskeleton, which undergoes rapid remodelling during neuronal migration^34^,^35^. Our results thus emphasize the importance of the balanced activity of pathways that regulate cytoskeleton dynamics in migrating cells. In agreement with our model of a pro-migratory function of Plexin B2 in cortical neurons, abnormal migration phenotypes have also been observed in the cerebellum and the rostral migratory stream of *Plexin B2* knockout animals^36^,^37^,^38^.

By using FRET imaging, we show that Plexin B2 stimulates RhoA activity in migrating cortical neurons, and that this regulation is essential for the migration of these cells, as the migratory defect of *Plexin B2*-silenced neurons is rescued when RhoA is overexpressed. Similar results have been reported for endothelial and breast cancer cells, where Plexin B1 induces cell migration via a RhoA-ROCK pathway^39^, suggesting that RhoA activation may represent a general mechanism by which Plexin B receptors promote the migration of various cell types. The recruitment of RhoA GEFs is the predominant mechanism by which Plexins activate RhoA in navigating growth cones^16^. A similar mechanism also operates in migrating cortical neurons, as preventing RhoGEF recruitment partially abolishes the pro-migratory activity of Plexin B2 and prevents RhoA activation upon Semaphorin stimulation. However, we show that RhoGEF recruitment is not the sole mechanism by which Plexin B2 activates RhoA. Plexin B2 also disrupts the interaction between Rnd3 and p190RhoGAP by competing with p190RhoGAP for Rnd3 binding, resulting in the activation of RhoA. In fact, Plexin B2 must both inactivate Rnd3 and recruit RhoGEFs to promote RhoA activity, as preventing Plexin B2 recruitment of RhoGEFs without interfering with its interaction with Rnd3 is not sufficient to stimulate RhoA activity.

Several studies have demonstrated that excessive RhoA activity (achieved through loss of the proneural gene *Neurog2* or loss of *Rnd* genes) arrests cortical neurons at different stages in their migration route, and that cell migration can be restored by reducing RhoA expression or blocking its activity^40^,^41^,^42^. Conversely, we have shown in this study that a reduced activity of RhoA also interferes with neuronal migration, as *Plexin B2*-silenced neurons that have reduced RhoA activity are stalled and overexpressing RhoA rescues their migration. Surprisingly, however, a recent study has shown that RhoA-deficient neurons migrate correctly in a wild-type environment^43^, suggesting that RhoA activity is not required cell autonomously for radial migration in the developing cerebral cortex. However, it is likely that other molecules can substitute for RhoA when the *RhoA* gene is deleted in neurons. Both RhoB and RhoC are expressed in the developing CP^44^, and RhoB has been shown to be strongly upregulated in the absence of RhoA^45^.

The fact that both inhibition and activation of RhoA are necessary for radial migration in the cortex (this work and refs 10, 46) is a striking illustration of the need for Rho GTPase activity to be tightly regulated. The multipronged regulation of RhoA may allow it to be simultaneously active in some parts of the cell and suppressed in other parts. The two RhoA inhibitors Rnd2 and Rnd3 are distributed in distinct subcellular compartments (endosomes and plasma membrane, respectively^10^), and are therefore likely to contribute to the differential regulation of RhoA within the cell. Similarly, Plexin B2 may stimulate RhoA activity only locally, such as in the proximal region of the leading process and at the rear of the cell, where active RhoA has been shown to be localized and essential for actin-based cellular contractility and nucleokinesis^35^. Localized activity of Plexin B2 may result from a restricted subcellular distribution of the receptor itself or from an extracellular gradient of a Semaphorin ligand. Several class 4 Semaphorins are expressed in the embryonic CP (Supplementary Fig. 5e) and may activate the Plexin B2-RhoA pathway in the leading process of cortical neurons to promote their directed migration.

In conclusion, we propose that the main role of the Ascl1-Rnd3 pathway is to maintain RhoA activity at a low level to provide the actin cytoskeleton with the plasticity required for efficient migration. Conversely, local Semaphorin-Plexin B2 signalling in the leading process or at the cell rear may activate RhoA and stimulate contractility by releasing Rnd3-mediated RhoA inhibition, and also by promoting Rho GEF-mediated RhoA activation. This work identifies a mechanism that fine-tunes RhoA activity in migrating neurons and will help better understand the pathogenesis of human neurodevelopmental disorders.

## Methods

### Plasmid constructs

*PlexinB1* shRNA (ID-3: 5′-GGAGTCATATTTCTGCTACTT-3′) and *PlexinB2* shRNA (ID-3: 5′-GGAGTCATATTTCTGCTACTT-3′) plasmids were purchased from SABiosciences. The control shRNA plasmid for PlexinB2 and PlexinB1 was provided by SABiosciences and contains the non-targeting sequence 5′-GGAATCTCATTCGATGCATAC-3′. For FRET experiments, *PlexinB2* shRNA and the relative control shRNA were cut with NdeI and AgeI to excise the green fluorescent protein (GFP). *Rnd2* and *Rnd3* shRNAs were obtained by cloning the following sequences into the shRNA vector, pCA-b-EGFPm5 silencer 3, (generous gift from Dr Vermeren): Rnd2, 5′-GGGCGAGATGCATAAGGAT-3′ (Ambion, ID 65909) and Rnd3, 5′-GCACATTAGTGGAACTCTC-3′ (Ambion, ID 166122) (refs 9, 10). For FRET experiments, EGFP in *Rnd3* shRNA and in the relative control shRNA plasmids was replaced by red fluorescent protein (RFP)^10^. *p190RhoGAP* shRNA (5′-GGCAGTTACTGACAGTCAA-3′) and the relative control (5′-GCGCGCTTTGTAGGATTCG-3′, Dharmacon) shRNA were cloned into pSuper_puro (Oligoengine). A shRNA-resistant PlexinB2 construct (pcDNA-VSV-PlexinB2*) was obtained by site-directed mutagenesis (QuickChange II Site-Directed Mutagenesis Kit, Stratagene) of a murine VSV-Plexin B2 expression plasmid^47^, using the following primers: forward, 5′-cgtgttctacaacgacactaaagtcgttttcttgtctcctgctgtc-3′ and reverse, 5′-gacagcaggagacaagaaaacgactttagtgtcgttgtagaacacg-3′. The underlined nucleotides identify the three silent mutations. PlexinB2 C-terminal deletion mutant was obtained by removing the last seven amino acids of PlexinB2* from murine pcDNA-VSV-PlexinB2* (ref. 20). The expression plasmid for human VSV-tagged PlexinB1 was a kind gift from Dr Luca Tamagnone^48^. The pcDNA-hRhoA was obtained from Missouri S&T cDNA Resource Center.

### *In utero* electroporation and tissue processing

*In vivo* genetic manipulation was performed by injecting the constructs of interest directly into mouse embryonic brains through *in utero* electroporation^17^,^49^. Mice were housed, bred and treated according to the Home Office guidelines, under the Animal Scientific procedure act 1986, and experiments were approved by the Home Office (project licence 707644). Before surgery, animals were injected with the analgesic drug buprenorphine (Vetergesic, Alstoe Ltd), and they were anaesthetized with isofluorane gas in oxygen carrier delivered by a Harvard Apparatus. Embryos were carefully exposed and the DNA was injected into the lateral ventricles of the brain and visualized by addition of the Fast-Green dye (Sigma, 1:20 ratio) into the DNA mix solution. DNA (1 μg μl^−1^, unless otherwise stated) was prepared in endotoxin-free conditions (Qiagen Maxi kit) and was injected using borosilicate pulled capillaries (1 × 0.58 mm, GC100-10, Harvard Apparatus) and a Femtojet microinjector (Eppendorf). To target the cortex, two 5 mm platinum electrodes (Tweezertrode 45-0489, BTX, Harvard Apparatus) were positioned onto the embryonic brains, and five electrical pulses at 30 V were applied across the uterine wall at 1 s intervals. To determine the amount of proliferating cells, pregnant mice were injected 1 day after electroporation with the thymidine analogue 5-ethynyl-2′-deoxyuridine EdU (Invitrogen) at the concentration of 30 μg per gram of animal. Mixed sex embryos were analysed at 1, 2 or 3 days after the electroporation. Mouse embryonic brains were fixed in 4% paraformaldheyde, cryoprotected in 20% sucrose/PBS and embedded in the OCT compound (VWR). A cryostat (Leica) was used to cut 14-μm-thick coronal sections of frozen embryonic brains. For immunostaining, paraformaldheyde-fixed sections were blocked for 30–45 min with 10% serum in 0.01% Triton X-100–PBS and incubated overnight at 4 °C with the following primary antibodies: chicken anti-GFP, 1:700 (Millipore, 06-896); mouse anti-ki67, 1:50 (BD Pharmingen) or treated with the EdU Click-iT kit (Life Technologies). The secondary antibodies, goat anti-chicken, 1:800 (Invitrogen) and goat anti-mouse, 1:500 (Jackson Laboratories), together with the nucleic acid marker TOTO-3 iodide, 1:1,000 (Invitrogen) were incubated with the sections for 1 h at room temperature. Images were acquired with a confocal microscope (Radiance 2100, Bio-Rad; or SP5, Leica) and quantified using the MetaMorph software (Molecular Devices). Statistical analysis was performed with the Prism software using an unpaired two-tailed Student’s *t*-test between control and knockdown condition. Multiple conditions were analysed by one-way analysis of variance (ANOVA) followed by the Bonferroni *post hoc* test. *P<0.05, **P<0.01, ***P<0.001 and ****P<0.0001.

### *In situ* hybridization

The riboprobes used to visualize the expression of *Rnd2* and *Rnd3* were previously described^9^,^10^. The RNA probes for *PlexinB1*, *PlexinB2* and *PlexinB3* were kindly provided by Dr Hannu Sariola^50^. RNA probes for *Sema4A*, *Sema4B*, *Sema4C*, *Sema4D*, *Sema4F* and *Sema4G* were previously described^47^. *In vitro* transcription was performed in the presence of digoxigenin (DIG)-marked nucleotides (Roche) under standard reaction conditions and the probes were purified using Amersham column. Non-radioactive *in situ* hybridizations were performed on 14-μm cryostat sections mounted on superfrost slides (Fisher). The sections were hybridized overnight at 70 °C with DIG-labelled RNA probes complementary to target mRNAs. To detect the riboprobes bound to the tissue, sections were incubated overnight at 4 °C with an anti-DIG antibody conjugated with alkaline phospatase. The signal was then revealed by adding nitro blue tetrazolium 5-bromo 4-chloro 3-indolyl phosphate (NBT/BCIP, Roche), the substrate of alkaline phosphatase (AP), to the sections.

### Fluorescence resonance energy transfer

Intramolecular probes for RhoA (pRaichu1293x) and R-Ras (pRaichu205x) have been used to determine the state activation of the two GTPases. The probes have been developed and kindly provided by Dr Matsuda^26^. The probe was used at the following concentrations: pRaichu1293x (RhoA probe) 0.25 μg μl^−1^ and pRaichu205x (R-Ras probe) 0.4 μg μl^−1^.

Images for FRET analysis were acquired with a Leica SP5 laser scanning confocal microscope and a × 40 numerical aperture 1.3 oil objective (for *in vivo* samples) and with a Zeiss LSM 510 META laser scanning confocal microscope and × 63 Plan Apochromat numerical aperture 1.4 Ph3 oil objective as specified (for *ex vivo* samples). Samples were analysed by acceptor photobleaching as follows. The cyan fluorescent protein (CFP) and yellow fluorescent protein (YFP) channels were excited using the 440 nm diode laser and the 514 nm argon line, respectively. The two emission channels were split using a 445-nm dichroic mirror, which was followed by a 475–525-nm band-pass filter for CFP and a 530-nm long-pass filter for YFP (Chroma). Pinholes were opened to give a depth of focus of 2 mm for each channel. Scanning was performed on a sequential line-by-line basis for each channel. The gain for each channel was set to ~75% of dynamic range (12-bit, 4,096 grey levels) and offsets set such that backgrounds were zero. Time-lapse mode was used to collect one pre-bleach image for each channel followed by bleaching with a minimum of 50 iterations of the 514-nm argon laser line at maximum power (to bleach YFP). A second post-bleach image was then collected for each channel. Control non-bleached areas were acquired for all samples in the same field of view as bleached cells to confirm specificity of FRET detection. Pre- and post-bleach CFP and YFP images were then imported into Image J for processing (AccpbFRET plugin^51^). Within this, all pixels were re-registered (using the Fast Hartley Transform algorithm) to correct for any shifts in the *x*–*y* plane, bleedthrough between donor and acceptor channels, and efficiency of acceptor photobleaching were accounted for using the equations detailed below. FRET efficiency was calculated based on the following formula:




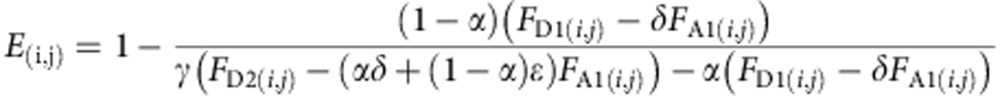




Where *F*_D1(*i*, *j*)_ and *F*_D2(*i*, *j*)_ are the donor fluorescence values of the pixel (*i*, *j*) before and after photobleaching the acceptor, and *F*_A1(*i*, *j*)_ the acceptor fluorescence for the same pixel before photobleaching. All *F* values were background corrected throughout. To correct for incomplete acceptor bleaching, the correction factor *α* is calculated as *α*=(*F*_A2(*i*, *j*)_/*F*_A1(*i*, *j*)_), where *F*_A2(*i*, *j*)_ and *F*_A1(*i*, *j*)_ are intensities in the acceptor channel in pixels above threshold of the donor- and acceptor-labelled sample, before and after photobleaching. The correction factor *γ* was implemented to correct for photobleaching of the donor during the image acquisition procedure can be calculated either as *γ*=(*F*_Dd1(*i*, *j*)_)/(*F*_Dd2(*i*, *j*)_), where *F*_Dd1(*i*, *j*)_ and *F*_Dd2(*i*, *j*)_ are donor fluorescence intensities of donor only (Dd) samples in pixels above threshold before and after photobleaching the acceptor. Correction for acceptor bleedthrough to the donor channel (correction factor *δ)* is calculated as *δ*=(*F*_Da1(*i*, *j*)_/*F*_Aa1(*i*, *j*)_), where *F*_Da1(*i*, *j*)_ and *F*_Aa1(*i*, *j*)_ are signals in the donor and acceptor channels in pixels above threshold of an acceptor only labelled sample, before photobleaching the acceptor. The correction factor *ɛ* accounts for any acceptor bleaching derived bleedthrough to the donor channel, and is calculated as *ɛ*=(*F*_Da2(*i*, *j*)_)/(*F*_Aa1(*i*, *j*)_), where *F*_Da2(*i*, *j*)_ and *F*_Aa1(*i*, *j*)_ are intensities in the donor and acceptor channels in pixels above threshold of an acceptor only labelled sample, before and after photobleaching.

### Cell culture

Mouse embryonic teratocarcinoma P19 and monkey kidney COS7 cells were cultured in high-glucose Dulbecco’s modified eagle medium (Invitrogen) supplemented with 10% fetal bovine serum, 2 mM glutamine and 1% penicillin/streptomycin, and they were incubated at 37 °C under 5% CO_2_ atmosphere. P19 cells were plated in 24-well plates and transfected with Lipofectamine 2000 reagent according to the manufacturer’s protocol (Invitrogen). Twenty-four hours post transfection (for *PlexinB1* and *PlexinB2* analysis) or 48 h post transfection (for *p190RhoGAP* analysis), cells were collected for protein or RNA extraction. COS7 cells were electroporated with expression constructs and analysed 24 h after transfection.

### Semaphorin production

Recombinant Semaphorin 4D was produced as a secreted protein fused with AP. To produce recombinant Semaphorin, human embryonic kidney HEK293T cells were transfected with Sema4D-AP in pcDNA3 vector^47^, using Fugene 6 reagent according to the manufacturer’s protocol (Promega) The supernatant of transfected cells was collected, filtered and concentrated 10-fold by Spin-X UF concentrators with a 100kDa cutoff (Millipore). Protein concentration was normalized to a range of 3–6 nM by measurement of the AP-activity using Alkaline Phosphatase Colorimetric Assay kit (Abcam).

### Real time PCR

*PlexinB1*, *PlexinB2* and *p190RhoGAP* mRNA expression was quantified by quantitative real-time PCR in control and shRNA-treated samples. RNAs were extracted from P19 cells using TRIzol reagent (Invitrogen), followed by a classical phenol/chloroform separation. Purified RNAs were then reverse-transcribed into cDNA with the Applied Biosystems kit. Real time PCR was performed with Taqman probes according to the manufacturer’s protocol (TaqManGene Expression Assays, catalogue number 4331182, Applied Biosystems). The 7500-system (Applied Biosystems) was used to run the PCR and analyse the data. *β-actin* was used for endogenous reference gene control and the values were normalized to control levels. Relative quantification was determined according to the DDc(t) method^52^. Data are presented as means±s.e.m. of normalized values from three independent experiments. Statistical analysis was performed with the Prism software using an unpaired two-tailed Student’s *t*-test between control and knockdown condition; **P<0.01 and ***P<0.001.

### *In vitro* co-immunoprecipitation and western blotting

Biochemical interaction between Rnds and Plexins was tested in P19 cells and COS7 cells. P19 cells were co-transfected with pCMV5-FLAG-Rnd3, pCMV5-FLAG-Rnd2, pcDNA-VSV-PlexinB1 and pcDNA-VSV-PlexinB2. Twenty-four hours after transfection, cells were lysed with RIPA-like lysis buffer (5 0 mM Tris–HCl, pH 8; 150 mM NaCl; 0.5% NP40 Igepal; 10% glycerol; protease and phosphatase inhibitor cocktails (Roche; Calbiochem)) and the insoluble material was removed by centrifugation at 13,000 *g* at 4 °C for 10 min. Anti-FLAG (cross-linked) agarose beads (M2 from Sigma) were first blocked in lysis buffer containing 0.2 mg ml^−1^ chicken egg albumin and 0.1 mg ml^−1^ insulin for 1 h at room temperature. Then the soluble cell lysate was incubated overnight at 4 °C with the beads. The day after, the beads were washed in lysis buffer and the proteins were eluted in Laemmli sample buffer, COS7 cells were co-transfected with pCMV5-FLAG-Rnd3 or pCMV5-FLAG-Rnd3^T55V^ and HA-PlexinB2 (cytoplasmic domain; a kind gift from Brad McColl), or with 5 μg of pCMV5-FLAG-Rnd3, pCMV-Myc-Δp190B (encoding amino acids 382–1,007 of p190RhoGAP-B)^27^ and increasing concentrations of pcDNA-VSV-PlexinB2 (0.5, 1, 2 and 5 μg) for the competition experiment^53^. Twenty-four hours after transfection, cell lysates were immunoprecipitated with mouse anti-FLAG M2 antibody (Sigma). Proteins were resolved by SDS–PAGE and analysed by immunoblotting, using the following primary antibodies: rabbit anti-VSV, 1:8,000 (Sigma, V4888); mouse anti-FLAG, 1:1,000 (Sigma, F1804); rabbit anti-FLAG 1:1,000 (Sigma, F7425), rabbit anti-actin, 1:1,000 (Sigma, A2066), mouse anti-GAPDH 1:10,000 (Millipore, MAB374), rat monoclonal anti-HA (3F10, Roche), rabbit anti-myc 1:500 (Santa Cruz, sc 789). For full blots see Supplementary Fig. 6.

## Additional information

**How to cite this article:** Azzarelli, R. *et al*. An antagonistic interaction between PlexinB2 and Rnd3 controls RhoA activity and cortical neuron migration. *Nat. Commun.* 5:3405 doi: 10.1038/ncomms4405 (2014).

## Supplementary Material

Supplementary Information Supplementary Figures 1-6
