# Supplementary material for: An antagonistic interaction between PlexinB2 and Rnd3 controls RhoA activity and cortical neuron migration
Source: Nat Commun. 2014 Feb 27;5:3405. doi: 10.1038/ncomms4405 (PMC3939360; doi:10.1038/ncomms4405)
Supplement: Supplementary Information — Supplementary Figures 1-6 [file ncomms4405-s1.pdf]

SUPPLEMENTARY INFORMATION

An antagonistic interaction between PlexinB2 and Rnd3 controls RhoA activity and cortical neuron migration

Roberta Azzarelli[1,4], Emilie Pacary[1,5,6], Ritu Garg[2], Patricia Garcez[1], Debbie van den Berg[1], Philippe Riou[2,7], Anne J. Ridley[2], Roland H. Friedel[3], Maddy Parsons[2], François Guillemot[1,@].

CONTENTS

Supplementary Figures 1 – 6

Supplementary Figure 1

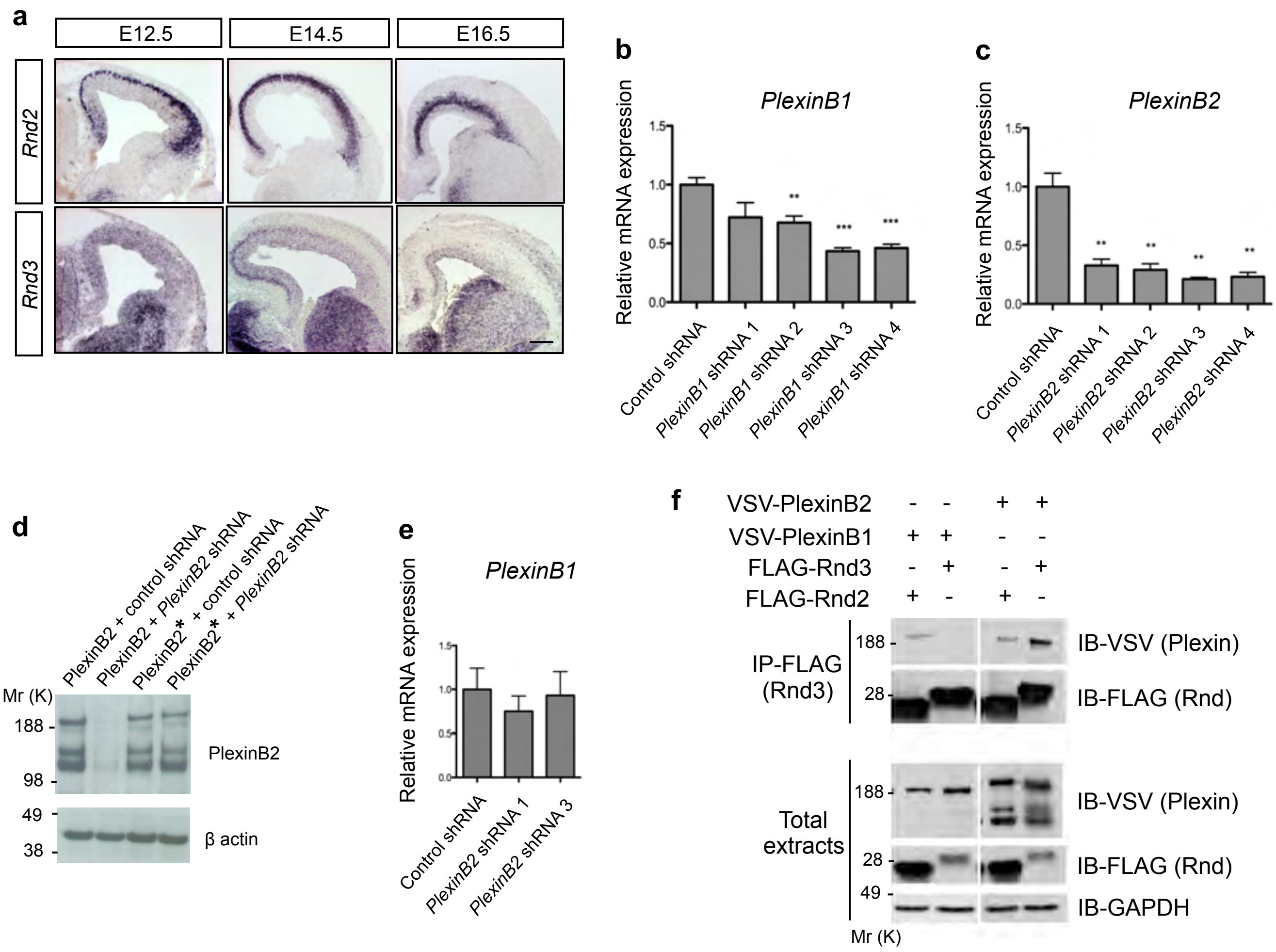

Supplementary Figure 1. Rnd3 is expressed in similar cortical domains as PlexinB2 and it specifically interacts with PlexinB2. (**a**) Expression profile of *Rnd* mRNAs. Distribution of *Rnd2* and *Rnd3* transcripts in coronal sections of the developing forebrain at different embryonic stages. Scale bar: 250 µm. (**b-e**) Efficiency and specificity of the shRNAs against Plexin B1 and Plexin B2. Relative quantification of *Plexin B1* (**b**) and *Plexin B2* (**c**) mRNA levels in P19 cells in the knockdown conditions, compared to the control. The values were normalised to *β-actin* level. Mean ± SEM of 3 independent experiments; Student's t-test, **$p<0.01$ ***$p<0.001$. The most efficient shRNAs (Plexin B1 shRNA 3 and Plexin B2 shRNA 3) were selected for subsequent analyses. (**d**) Western blot analysis of Plexin B2 protein expression in P19 cells transfected with different constructs, as indicated, showing that the 3 silent mutations introduced in PlexinB2 cDNA render it resistant to the shRNA. (**e**) Relative quantification of Plexin B1 mRNA levels following Plexin B2 knockdown using two distinct shRNAs. Mean ± SEM of 3 independent experiments. The application of the student's t-test revealed no statistically significant differences between *Plexin B2* shRNA-treated samples and control, indicating that PlexinB2 shRNA does not alter PlexinB1 expression. (**f**) Co-immunoprecipitation experiments between distinct PlexinB and Rnd members demonstrate that while PlexinB2 binds to both Rnd2 and Rnd3, PlexinB1 does not interact with Rnd3. COS7cells were transfected with VSV-*Plexins* (*PlexinB1* or *PlexinB2*) alone or in combination with FLAG-*Rnds* (*Rnd2* or *Rnd3*), as indicated. The lysates were immunoprecipitated with anti-FLAG antibody and immunoblotted with anti-VSV or anti-FLAG antibodies.

Supplementary Figure 2

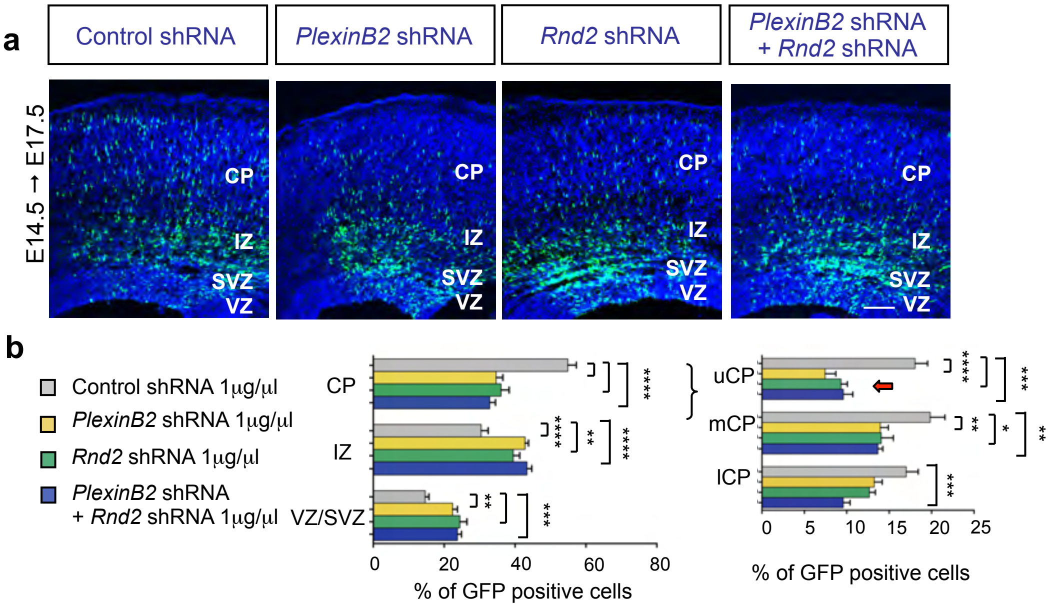

Supplementary Figure 2. Plexin B2 and Rnd2 do not interact genetically. (**a**) Double knockdown of *Rnd2* and *Plexin B2* does not ameliorate the migration defects induced by single gene silencing. Scale bar: 200 μm. (**b**) The red arrow in the quantification graph indicates that the percentage of neurons reaching the upper CP (uCP) is similar in single and double knockdowns. Mean ± SEM from 6 sections prepared from 3 different experiments ; One way ANOVA followed by Bonferroni post-hoc test; *p<0.05, **p<0.01, ***p<0.001, ****p<0.0001.

Supplementary Figure 3

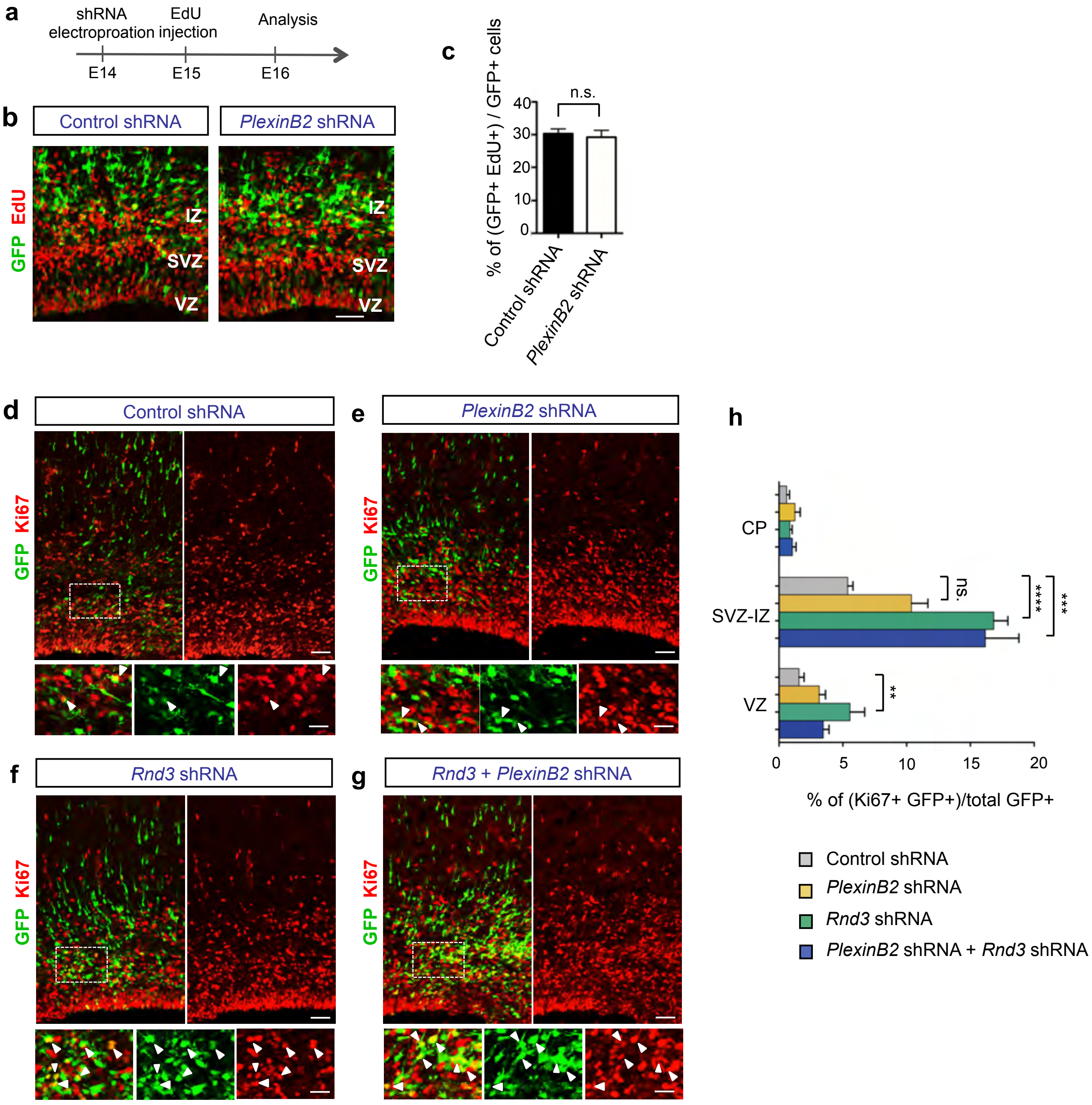

Supplementary Figure 3. Rnd3 controls cell proliferation in a Plexin B2-independent manner. (**a-c**) PlexinB2 does not influence the proliferation of cortical progenitors. (**a**) Time line of the experiment. (**b**) E16.5 cortices electroporated at E14.5 with control shRNA or PlexinB2 shRNA and immunostained for GFP (green) and EdU (red). Scale bar: 100 µm. (**c**) Quantification 2 days after electroporation of the percentage of electroporated cells that are EdU positive. Mean ± s.e.m from at least 6 sections prepared from 3 embryos obtained from 3 litters. The application of the student's t test revealed no statistically significant differences (n.s., not significant) between the PlexinB2 shRNA-treated samples and the control. (**d-g**) E17.5 cortices electroporated at E14.5 with different shRNAs as indicated and immunostained for GFP (green) and Ki67 (red). White arrowheads in insets indicate double positive cells. Scale bar: 50 µm and 20 µm (insets). (**h**) Quantification of the percentage of electroporated cells that are Ki67-positive. Mean ± s.e.m from at least 6 sections prepared from 3 embryos obtained from 3 litters. One way ANOVA followed by Bonferroni post-hoc test; **p<0.01, ***p<0.001, ****p<0.0001 n.s., not significant.

Supplementary Figure 4

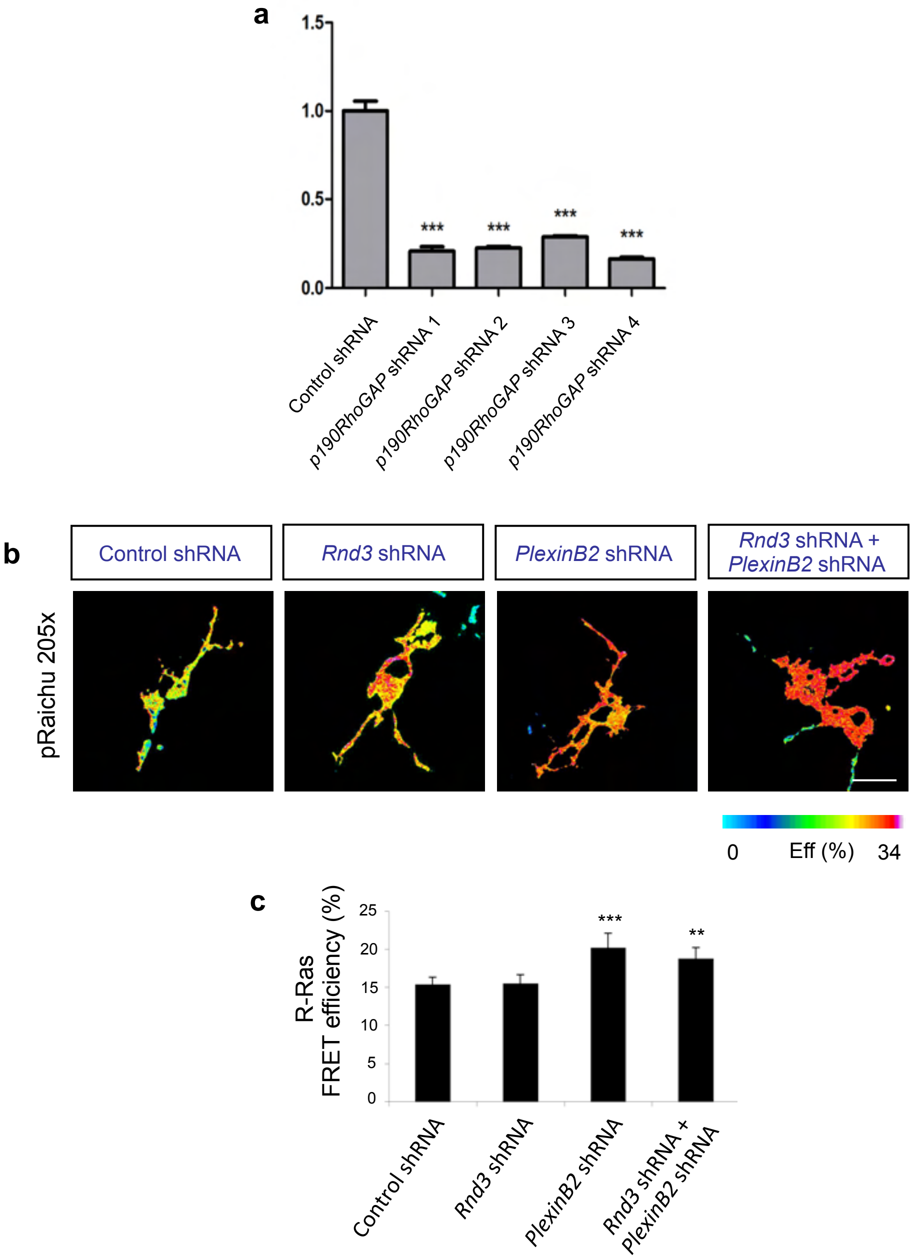

Supplementary Figure 4. PlexinB2 inhibits Rnd3 activation of p190RhoGAP, rather than Rnd3 controlling the R-Ras GAP activity of PlexinB2.

(**a**) Efficiency of p190RhoGAP shRNA. Relative quantification of *p190RhoGAP* mRNA levels in P19 cells in the knockdown conditions, compared to the control. The values were normalised to *β-actin* level. Mean ± SEM of 3 independent experiments; Student's t-test, ***p<0.001. The shRNA number 2 was selected for subsequent analysis. (**b**) PlexinB2 and Rnd3 regulation of R-Ras activity. *In vitro* FRET analysis of R-Ras activity in dissociated cortical cells in culture, 2 days after the electroporation of the constructs indicated. Scale bar: 10 μm (right panel). (**c**) Mean ± SEM; (n>20 cells for each condition, from 3 independent experiments; t-test: **p < 0.01, ***p < 0.001 compared to control). In contrast to *PlexinB2*, *Rnd3* silencing does not affect the levels of R-Ras-GTP.

Supplementary Figure 5

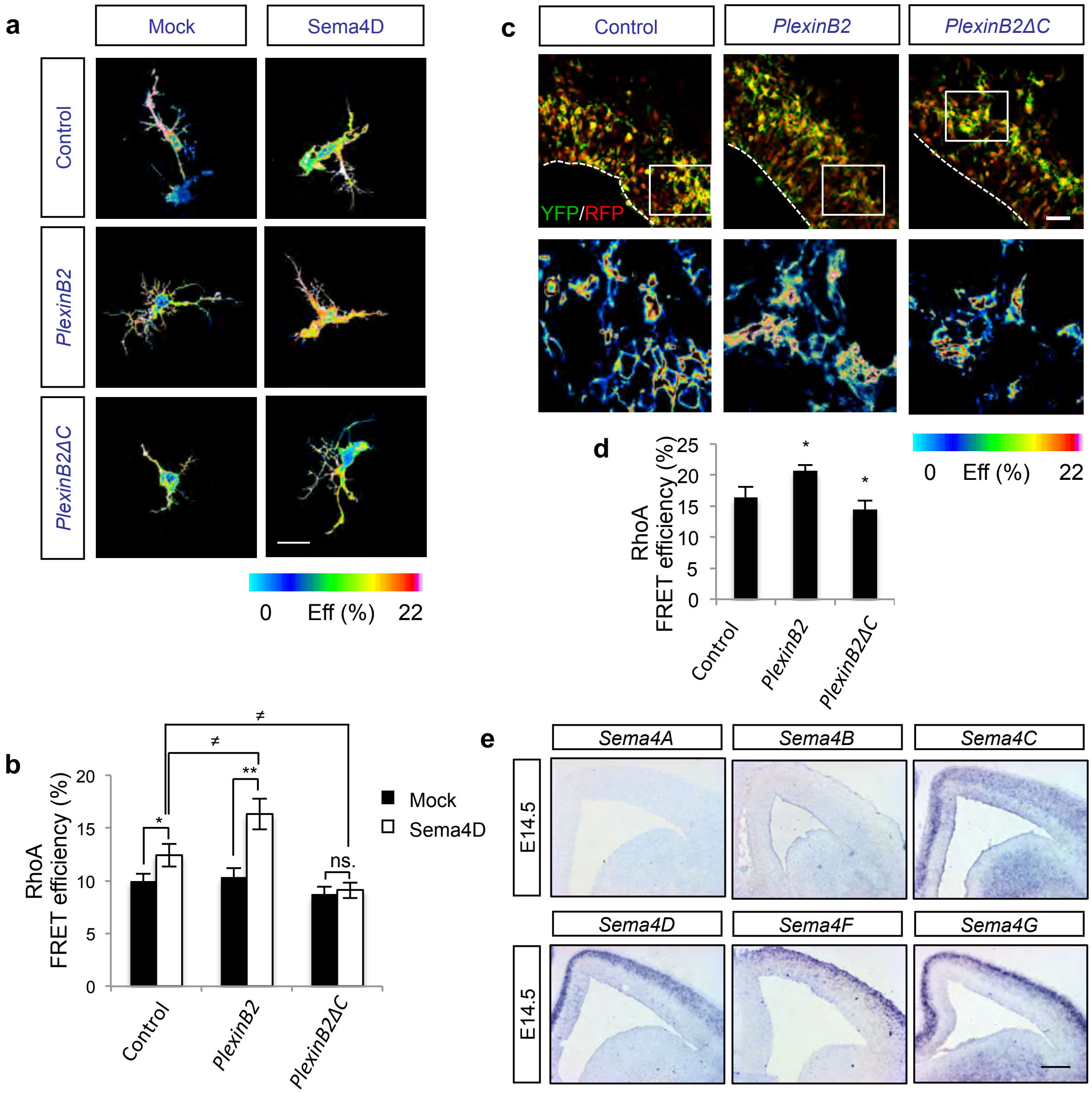

Supplementary Figure 5. PlexinB2 C-terminal deletion mutant loses the ability to induce RhoA activity *in vitro* after Semaphorin stimulation and *in vivo*. (**a**) *In vitro* FRET analysis of RhoA activity in dissociated cortical cells in culture, 2 days after PlexinB2 or PlexinB2 C-terminal deletion mutant (PlexinB2 ΔC) overexpression. Neurons have been treated with alkaline phosphatase (AP) (mock, left panels) or recombinant Semaphorin 4D-AP fusion protein (3-6nM) (right panels) for 45 minutes. Images are in pseudocolors, which represent the FRET efficiency. Scale bar: 10 μm (**b**) Mean ± SEM; (n>12 cells for each condition, from 3 independent experiments; t-test: *p < 0.05 **p < 0.01 compared to untreated cells in each condition, ≠ p < 0.05 between Plexin B2 overexpressing cells and control cells treated with Sema4D). (**c**) *In vivo* FRET analysis of RhoA activity performed on cortical slices electroporated with control empty vector or vectors overexpressing PlexinB2 full length or PlexinB2 ΔC. The analysis has been performed one day after electroporation in the regions of the upper IZ and lower CP. Upper panels show the YFP signal from the FRET probe and the RFP signal that marks electroporated cells (Scale bar: 50 μm). Lower panels show FRET efficiency. (**d**) Mean ± SEM (n>13 areas analyzed for each condition, deriving from 3 different embryos from 3 different litters; t-test *p < 0.05 compared to control) (**e**) Expression profile of class 4 Semaphorins. Coronal sections of embryonic murine brains at E14.5 were processed for in situ hybridization with riboprobes specific for *Sema4A, 4B, 4C, 4D, 4F* and *4G*. *Sema4A* and *4B* are not detectable at this developmental stage, whereas the other Semaphorins are expressed in the CP. Scale bar: 250 μm

Supplementary Figure 6

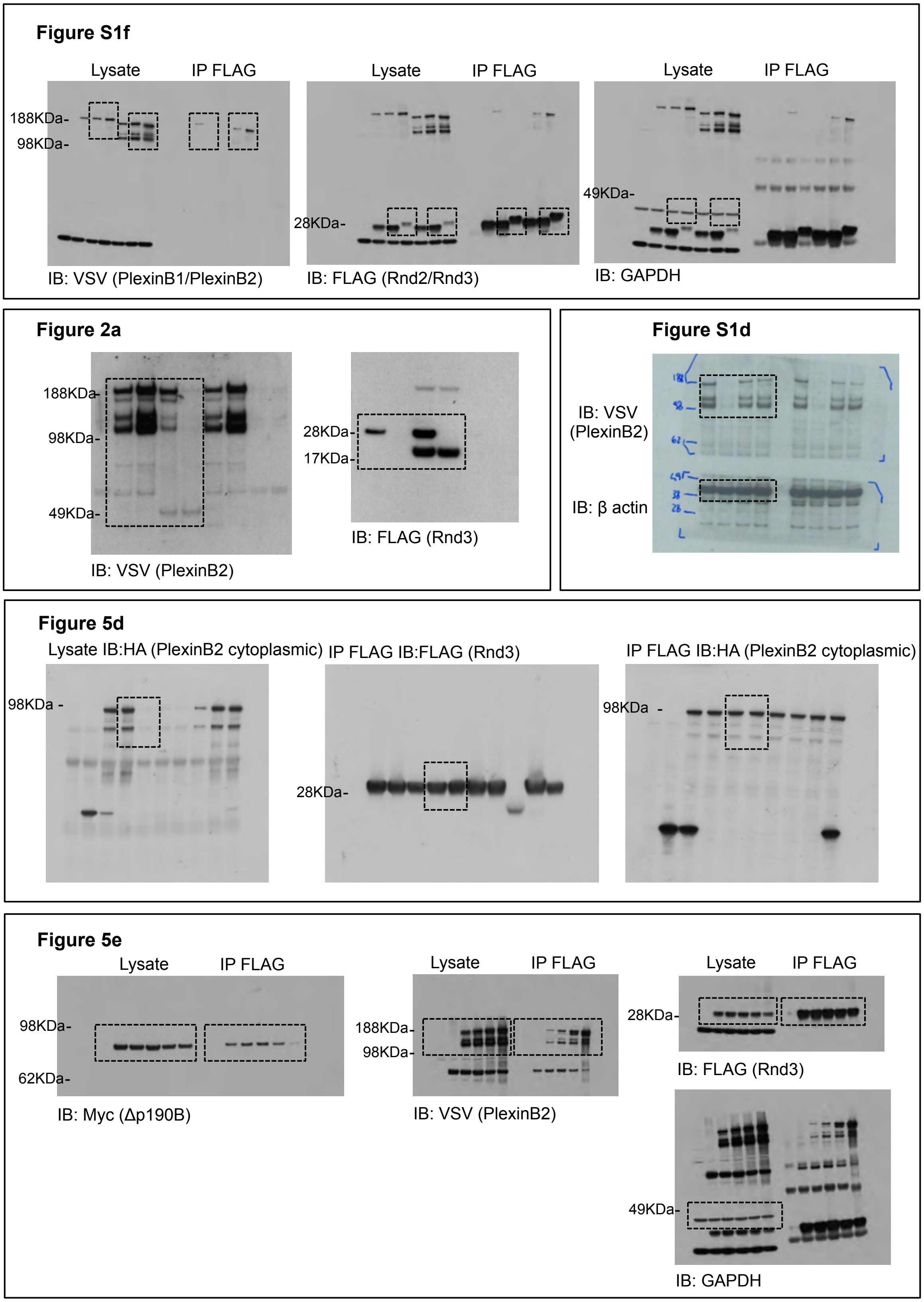

Supplementary Figure 6. Full blots from Figures 2a, 5d, 5e and supplementary figures 1d and 1f .
